# A combined behavioural economics- and simulation-based medical education to promote effectiveness among medical residents in coping with workplace violence in Northern China: a quasi-experimental study

**DOI:** 10.1186/s12889-022-13497-y

**Published:** 2022-06-01

**Authors:** Chao Liu, Weijing Liu, Mingli Jiao, Ye Li, Gangyu Zhang, Lifeng Wei, Shuang Zhou, Yuanheng Li, Zhuowa Sha, Yanhua Hao, Qunhong Wu

**Affiliations:** 1 Department of Health, Policy and Hospital Management, School of Public Health, Harbin Medical University, Harbin, 150081 China; 2 Department of Internal Medicine, Heilongjiang Academy of Chinese Medicine Science, Harbin, 150081 China; 3 Department of Social Medicine, School of Public Health, Harbin Medical University, Harbin, 150081 China

**Keywords:** Behavioural economics, Workplace violence, Simulation, Medical resident

## Abstract

**Background:**

Workplace violence is internationally recognised as a major concern for the workforce, which entails serious consequences, and research shows that medical residents are more likely than other doctors to experience violence in the workplace. This study first examines the effectiveness of simulation-based medical education, and then simulation-based medical education combined with behavioural economics as interventions in medical residents' perception of, attitude toward, and self-efficacy in coping with violence in the workplace.

**Methods:**

A quasi-experimental design was used, 190 participants were randomised into three study groups to respectively test the effect of simulation-based medical education only and simulation-based medical education plus behavioural economics interventions, compared with a control group. Data were obtained from structured questionnaires, including (1) a perception of aggression scale, a management of aggression and violence attitude scale, a general self-efficacy scale, and (2) socio-demographic characteristics.

**Results:**

The results show that the scores attained by simulation-based medical education (SBME) and simulation-based medical education combined with behavioural economics (SBME + BE) interventions for perception, attitude, and self-efficacy were significantly higher than those in the control group (*p* < .01). The SBME + BE group recorded a greater improvement in perception, which could be ascribed to the behavioural economics effect. Furthermore, the higher perception of workplace violence is correlated with single residents and those with more work experience, prior experiences of violence in the workplace, and training related to workplace violence. A higher positive correlation of workplace violence was recorded by female and widowed residents,and a higher level of self-efficacy related to violence in the workplace correlated with male, widowed,and senior (third-year) residents.

**Conclusions:**

This study contributes important evidence regarding changes in the perception, attitude, and self-efficacy of subjects following both the SBME + BE and SBME interventions among medical residents in coping with workplace violence, the biggest perception change having been recorded after the SBME + BE intervention, which can be explained by the inclusion of behavioural economics.

**Supplementary Information:**

The online version contains supplementary material available at 10.1186/s12889-022-13497-y.

## Background

Workplace violence (WPV) is described by the World Health Organization (WHO) as deliberate physical, psychological, sexual, and other acts against someone at work that may risk his/her health or even result in death [1]. It is recognized internationally as a major workforce concern [2] that causes serious consequences to clinicians, including mental health problems, insomnia, work stress, job dissatisfaction, decreased quality of health service, job transfers, and even resignations [3–6]. In the past two decades, a growing number of WPV incidents against clinicians has been reported in China, with detrimental effects on the medical community and increased rates of clinicians turnover [7, 8]. Further, violence against clinicians has grown continuously during the COVID-19 pandemic [9, 10].

Medical residents who perform basic medical work are more likely than other doctors to experience WPV [11] as they have little experience with such volatile encounters, spend more time with patients and the latter’s relatives than any other doctor, and are vulnerable to worker-to-worker violence from senior doctors, nurses, and other healthcare workers [12–14]. It is noteworthy that WPV against medical residents is underreported [15]. Several studies showed that WPV against medical residents had been reported in the United States [16, 17], Canada [18], China [19], India [20], Syria [21], Turkey [22], Romania [23], Peru [11], and Uganda [24]. Many studies highlighted the need for methods to ensure the safety of healthcare workers and proposed interventions to address the problem [25, 26]. It is therefore evident that the prevention of WPV and maintaining medical residents’ effectiveness are becoming pressing problems. Given the aforesaid, the International Labour Organization has established new global standards aimed at ending violence and harassment at the workplace [27].

Previous studies proposed the following strategies, based on their findings, to reduce the incidence of WPV: by eliminating risk factors in the work environment [28], proper training [29], and simulation-based medical education (SBME) [30]. In particular, SMBE is considered one of the most effective clinical teaching strategies as it provides a realistic but safe environment to address WPV-related issues [31]. More importantly, it could help healthcare workers learn to recognise risks, defend themselves, and become familiar with the process of dealing with possible conflicts. Previous studies show that SBME improves perception and confidence regarding WPV [32, 33], reduces the incidence of violence [28, 33, 34], and improves knowledge, skills, ability, and preparedness concerning WPV [35, 36]. However, research that conducted comprehensive analyses of the effectiveness of WPV education and intervention strategies among medical residents in China remains limited.

The previous studies selected the perception and confidence of WPV among the outcome measures of the SMBE study [32, 33]. As we know,WPV is closely related to physical and mental pain [37]. Psychological research shows that the cognition, attitude, and willpower towards pain are closely related to pain tolerance and endurance [38]. Therefore, the study chooses cognition, attitude and self-efficacy as the effectiveness of WPV education and intervention strategies.

Behavioural economics (BE) blends economics with psychology and acknowledges that people often do not act rationally in the economic sense. It provides an expanded set of tools for understanding and influencing behaviour, compared to traditional economic theory [39–41]. There is increasing interest in research on the BE of violence [42], with multiple applications focusing on intimate violence [43], doctor-patient conflicts [44], crime prevention through environmental design [45], and reducing fear and stigma [46] in the healthcare sector, but it has not been extended to include WPV. Moreover, although an association between BE and violence prevention is suggested by some quantitative data and applying BE in medical education has been mooted [47], little is known about the BE and consequences of WPV against medical residents. Hence, we planned this study to determine the effect of SBME + BE and SBME-only, respectively, in considering a WPV prevention programme to improve the perception of, attitude toward, and self-efficacy of WPV victims and related factors among medical residents at Harbin Medical University. Our view is that residents may benefit from further education on how to cope with WPV, and that this study will assist them with their clinical practice and competency in WPV prevention.

### Theoretical framework

**Fig. 1:**
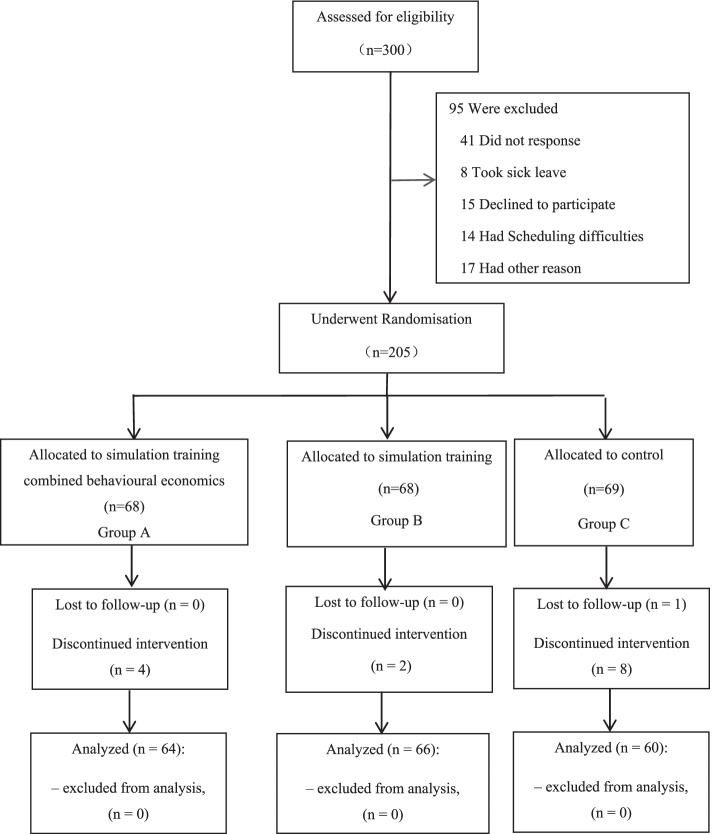
Flow diagram of enrollment

This study constructed a BE theory theoretical framework combined Haddon matrix to predict, identify and evaluate the risk of doctor-patient decision-making, and to improve the response and management of WPV. Moreover, this study described the role of peak-end rule, framing effect and loss aversion in WPV prevention in behavioral economics theory (see Fig. 1).

The peak-end rule represents the critical moments of an experience and dominates and dominate our good or bad feelings about it [48]. It is well known that patients’ dissatisfaction with medical services is one of the reasons for WPV. Previous studies found that if the patient felt satisfied with the peak and end experiences of the healthcare service, they were more likely to feel satisfied with the entire service process [49]. Therefore, improving patient satisfaction and reducing patients’ decision-making burden during the treatment process at peak and final moments can improve overall satisfaction and reduce the incidence of WPV [50].

Loss aversion refers to the fact that patients weigh the gains and losses of health services with the losses being 2.5 times the weight of the gains [51]. Previous studies have found that compliance with medical advice is closely related to the disease prognosis. However, However, perceived losses of freedom in personal habits, and financial loss, causes evident aversion and obstacles to compliance with medical advice, leading to poor treatment effects and may easily induce WPV [52]. Previous studies used loss aversion to motivate patients to make rehabilitation training decisions, and improved health monitoring and physical activity of overweight patients at a lower economic cost [53], thus avoiding the occurrence of WPV caused by poor rehabilitation results.

The framing effect suggests that different descriptions of logically equivalent information lead to different decision judgments [54]. A lack of communication between doctors and patients is an important cause of WPV. The framing effect can be used effectively in doctor-patient communication [55]. Previous studies have found that doctors who used the framing effect to communicate with women with average and higher levels of perceived susceptibility to breast cancer, found that it promoted breast cancer screenings [56]. Therefore, when communicating with patients, a patient-friendly information framework should be adopted to increase doctor-patient interaction and avoid WPV due to inadequate communication.

The Haddon matrix is a framework of injury prevention and control proposed by William Haddon [57]. Carol Runyan introduced the Haddon matrix framework and presented intervention strategies and recommendations for WPV prevention. The use of the Haddon matrix has contributed considerably to the understanding of WPV occurrence. It has two axes. The first axis, includes the elements of the epidemiological triad, host (healthcare workers), vector (patients), and environment, and likens injury to a disease similar to an infection or cancer. The second axis on that grid includes three time intervals pre-event, during-event, and post-event [58]. The importance of including these intervals was that injury was conceptualised as an event predictable within time, and as such, amenable to study within populations, and to WPV prevention. This study constructed a conceptual model of WPV prevention, based on the theoretical framework of behavioral economics and the introduction of Haddon matrix framework.

This study reviewed the literature on recommendations for interventions, discussed the implement the intervention strategies by simulation based medical education and BE education combined the Haddon matrix to address workplace violence.

## Methods

### Study design, sampling, and data collection

**Fig. 2:**
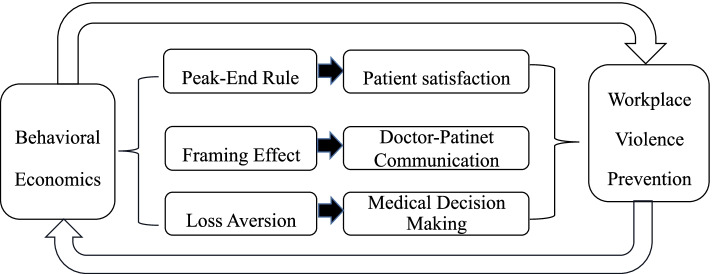
Behavioural economics approaches to workplace violence prevention

We carried out a quasi-experimental study and single-blind study between December 2020 and January 2021 at Harbin Medical University, Heilongjiang Province, China. All medical residents who had completed the three-month residency training and who displayed no intention to suspend their studies or leave the University after nearly six months were considered eligible for the study. Medical residents from departments with a low incidences of workplace violence (endocrinology, haematology, and laboratories) and infectious departments closely related to COVID-19 were excluded from the study. A flow diagram depicting enrollment, randomised assessment for eligibility, and follow-up of study participants, is illustrated in Fig. 2.

### Sample size and sample procedure

The statistical software G* Power was used to calculate the sample (https://stats.idre.ucla.edu/other/gpower/one-way-anova-power-analysis/). In this study, effect size = 0.25, maximum allowable error (β) = 0.2, *p-*value (α) = 0.05 were set for three groups. The total number was calculated at 177, and the proportion of attrition and was taken as 10%. After adjusting for the number of non-responses, the final number of participants was 195. Stratified random sampling was carried out to select the participants, and block randomisation was adopted for a 1:1:1 random grouping into the following groups: Control, SBME, and SBME + BE. The participants completed both the paper and online questionnaires before and immediately after the intervention.

### Intervention

In this study, interventions were constructed based on the two dimensions of the Haddon matrix, the epidemiological triad dimension and the time dimension [59]. In the epidemiological dimension, WPV prevention intervention focuses on improving the ability of doctors to cope with WPV and predicting the possible factors of violence caused by patients. In the time dimension, pre-event intervention aims to prevent a violent episode from occurring, during event intervention aims to control the WPV, and post-event intervention aims to minimise the damage of injuries resulting from assaults. Finally, the effectiveness of the residents' response to WPV could be used as a third dimension to strengthen the advantages of the Haddon matrix in an intervention [60].

The control group received training on ‘workplace violence prevention’, spanning 3 h and 20 min, which was based on systematic reviews [28, 58, 61, 62]. Lectures were given by senior professors with experience in WPV. The SBME group received 4 h and 40 min’ worth of training on ‘simulation education on workplace violence training’, which was based on the systematic reviews and augmented by senior professors with experience of WPV focus group interviews [34, 35, 63]. This simulation training was developed using a six-step approach [64], and lectures were given by senior professors with experience of WPV in emergency departments and intensive care units. Each case was trained for 70 min per week for five consecutive weeks, and each teacher was assigned to present one training session. The participants, who acted as patients, nurses, doctors, and family members, conducted a simulation drill, which was captured on film and replayed. The SBME + BE group received 3 h’ training on ‘behavioural economics of workplace violence education’ and ‘simulation education on workplace violence’. BE studies have shown that the peak-end rule [49], loss aversion [65], and framing effects [66] directly impact on patient satisfaction, medical decision-making, and doctor-patient communication, which are closely related to the occurrence of WPV [67, 68]. Therefore, the ‘behavioural economics education’ is designed according to relevant literature research and actual reduce the incidence of WPV while improving health outcomes. The details of the training courses are shown in Table 1.

**Table 1:** Workplace violence interventions combined Haddon matrix

Group	Item	Interventions	Methodology	Duration
Group A Group B	Pre-	Scenario 1 Human relation skills training (e.g., communication, team building, problem solving, diversity and conflict resolution)	Explanation and demonstration in the clinical Skill Center Video watching Simulation training	280 min
	During	Scenario 2 Workplace violence prevention training (e.g., verbal degrade and physical control during the inpatient/ outpatient/ICU treatment) Scenario 3 Evacuation skills training(Report workplace violence, evacuate from a safe route)		
	Post-	Scenario 4 Disposal training for injured medical workers (e.g., report injuries and transfer injured medical workers for medical treatment) Video playback and scenario review discussion		
Group A	Pre-	The introduction of behavioral economics and review behavioral economics of violence	Explanation in the clinical Skill Center	180 min
	During	Behavioral economics approaches and strategies for preventing workplace violence		
	Post-	Behavior intervention used in workplace violence prevention and discussion		
Group C	Pre-	The prevalence, causes and risk identification of workplace violence	Explanation in the classroom Video watching	200 min
	During	Review workplace violence prevention and strategies (including how to deal with the perpetrator, anger management, self-protection)		
	Post-	Review policy, medical liability, and legal knowledge related to workplace violence		

### Outcome variables

Perception, attitude, and self-efficacy were classified as the outcome variables based on the psychological research, which provided approaches for a BE intervention. Perceptions of WPV were assessed by using the validated Chinese version of the perception of aggression scale (POAS), which comprised 12 items [69]. Examples of characteristic items are: ‘aggression is an unpleasant and repulsive behaviour’, ‘aggression is unnecessary and unacceptable’, and ‘aggression hurts others mentally or physically’. All items were rated on a five-point Likert scale (1 = strongly disagree, 5 = strongly agree). A higher score indicates a more positive view and higher tolerance towards patient aggression. Attitude was assessed by using the validated Chinese version of the management of aggression and violence attitude scale (MAVAS), which comprised 27 items [70, 71]. The MAVAS was scored on a 5-point Likert scale (1 = strongly disagree, 5 = strongly agree). Higher scores could indicate higher levels of the respondents’ agreement with the items regarding the specific explanatory model of violence. Self-efficacy was assessed by using the validated Chinese version of the general self-efficacy scale (GSES) [72]. It consists of 10 items, arranged on a 4-point rating scale. Higher scores suggest higher self-efficacy and stronger willpower when dealing with workplace violence [73]. The above three scales have good reliability and are widely used in workplace violence research for doctors, nurses, and other healthcare workers. Details about the questionnaire in this study are presented in Additional file 1.

### Independent variables

Socio-demographic factors included age, day shifts per week, night shifts per month, sex, marital status, year of residence, department, work experience, working hours per day, workplace violence concerns, workplace violence training, reports of workplace violence, physical violence witnesses, exposure to verbal violence, exposure to physical violence, and exposure to sexual harassment. These data were collected to identify the risk factors related to workplace violence [74].

### Statistical analyses

Descriptive analyses were performed for category variables such as sex, postgraduate year, marital status, work experience, and workplace violence experience; these variables were described by frequency distribution and percentage. Continuous variables such as age were described by mean and standard deviation (STD) or median and quartile. ANOVA, non-parametric test or chi-square/Fisher tests were used to analyse the comparison between the groups, and Bonferroni’s and Dunnett’s tests were used to analyse the pairwise comparisons. Differences between the pre- and post-test performance of perception, attitude, and self-efficacy pertaining to workplace violence were compared using generalised estimating equations (GEE). Data analyses were performed by using the software SPSS 25.0.

## Results

### Participants’ socio-demographic characteristics

The 205 participants were recruited through telephonic inquiries and resident referrals, and 68.33% of eligible residents agreed to participate in the study. Overall, 190 participants who matched the inclusion criteria completed the study (Fig. 2). Table 2 displays the characteristics of the enrolled participants. The median age of the study participants was 25 years. The most common participant categories were female (59.47%), single (60.94%), and first-year residents (41.58%). Most of the participants were from internal medicine (41.58%), followed by surgical (33.1%), neurology (13.68%), radiology (10.53%), gynaecology (5.79%), and paediatrics (2.11%). Many participants had 12–24 months of work experience (48.95%). The major work-related concerns recorded by participants were workload (76.32%) and concern about workplace violence (89.47%). Nearly half (47.89%) of the participants had received workplace violence training, but only 63 (33.16%) participants had reported violence in the workplace. Witnesses of physical violence were found in 54 (28.42%) of participants’ cases. The participants who had been exposed to verbal violence, physical violence, and sexual harassment were 53.68%, 3.68%, and 22.63% respectively.

**Table 2:** Socio-demographic characteristics of medical residents

Characteristics	Overall *n* = 190	Group A *n* = 64	Group B *n* = 66	Group C *n* = 60	Z or χ^2^	p
	median(quartile) n, (%)	n,(%)	mean ± sd n,(%)	mean ± sd n,(%)		
Age	25(24,26)	25(25,26)	24(24,25)	25(24,26)	1.682	.190
Sex						
Male	77(40.53)	25(39.06)	26(39.39)	26(43.33)	0.288	.865
Female	113(59.47)	39(60.94)	40(60.61)	34(56.67)		
Marital status						
Single	175(92.11)	59(92.19)	60(90.91)	56(93.33)	3.489	.811
Married	5(2.63)	3(4.69)	1(1.52)	1(1.67)		
Unmarried cohabitation	7(3.68)	1(1.56)	4(6.05)	2(3.33)		
Widowed	3(1.58)	1(1.56)	1(1.52)	1(1.67)		
Resident year						
PGY1	79(41.58)	24(37.5)	30(45.46)	25(41.67)	1.538	.819
PGY2	68(35.79)	24(37.5)	24(36.36)	20(33.33)		
PGY3	43(22.63)	16(25)	12(18.18)	15(25)		
Department						
Pediatrics	4(2.11)	2(3.13)	2(3.03)	0(0)	3.990	.961
Obstetrics-Gynecology	11(5.79)	5(7.81)	2(3.03)	4(6.67)		
Internal medicine	79(41.58)	25(39.06)	30(45.45)	24(40)		
Neurology	26(13.68)	10(15.63)	7(10.61)	9(15)		
Surgical	50(26.32)	16(25)	19(28.79)	15(25)		
Radiology	20(10.53)	6(9.38)	6(9.09)	8(13.33)		
Work experience						
< 6 months	14(7.37)	5(7.81)	4(6.06)	5(8.33)	0.434	.517
6–12 months	65(34.21)	19(29.69)	26(39.39)	20(33.34)		
12–24 months	93(48.95)	31(48.44)	33(50)	29(48.33)		
> 24 months	18(9.47)	9(14.06)	3(4.55)	6(10)		
Working hours per day						
< 8 h	45(23.68)	16(25)	16(24.24)	13(21.67)	0.428	0.981
8–12 h	128(67.37)	42(65.63)	45(68.18)	41(68.33)		
> 12 h	17(8.95)	6(9.37)	5(7.58)	6(10)		
Workplace violence concern						
Absolutely not worried	20(10.53)	8(12.5)	8(12.12)	4(6.67)	8.544	.382
A little worried	75(39.47)	31(48.43)	18(27.27)	26(43.33)		
Moderately worried	48(25.26)	13(20.31)	20(30.3)	15(25)		
Worried	26(13.68)	6(9.38)	11(16.67)	9(15)		
Very worried	21(11.05)	6(9.38)	9(13.64)	6(10)		
Workplace violence report						
Yes	63(33.16)	25(39.06)	21(31.82)	17(28.33)	1.691	.429
No	127(66.84)	39(60.94)	45(68.18)	43(71.67)		
Workplace violence training						
Yes	91(47.89)	36(56.25)	34(51.52)	21(35)	6.134	.047
No	99(52.11)	28(43.75)	32(48.48)	39(65)		
Physical violence witness						
Yes	54(28.42)	18(28.13)	19(28.79)	17(28.33)	0.007	.996
No	136(71.58)	46(71.87)	47(71.21)	43(71.67)		
Verbal violence exposure						
Yes	104(53.68)	31(48.44)	38(57.58)	33(55)	1.152	.562
No	86(46.32)	33(51.56)	28(42.42)	27(45)		
Physical violence exposure						
Yes	7(3.68)	2(3.13)	2(3.03)	3(5)	0.549	.797
No	183(96.32)	62(96.87)	64(96.97)	57(95)		
Sexual harassment exposure						
Yes	43(22.63)	11(17.19)	18(27.27)	14(23.33)	1.912	.383
No	147(77.37)	53(82.81)	48(72.73)	46(76.67)		

The results indicate no significant differences among residents’ socio-demographic characteristics between groups A, B, and C. Although workplace violence training reflected significant differences among the three resident groups (*p* = 0.047), there were no significant differences in pairwise comparisons (*p* > 0.0167). The results show that there was homogeneity among the groups, which meets the basic condition of intervention.

To test for perception, attitude, and self-efficacy pertaining to workplace violence, all three groups received pre-and post-test questionnaires. To analyse the effect of simulation training and BE teaching after the intervention, we measured perception, attitude, and self-efficacy related to WPV by using POAS, MAVAS, and GSES. These results indicated no significant difference in the pre-test scores among the three groups for the perception, attitude, and self-efficacy of WPV, as *p* > 0.05 (see Table 3).

**Table 3:** Comparison of pre- and post-test of POAS, MAVAS and GSES

Item	Pre-Test	F	p	Post-Test	F	p
POAS						
Group A	37.59 ± 5.31	0.221	.801	44.49 ± 6.52^ac^	6.731	.002
Group B	37.08 ± 7.05			41.75 ± 7.18^b^		
Group C	37.75 ± 5.38			38.18 ± 5.43		
MAVAS						
Group A	88.47 ± 12.64	0.087	.916	96.49 ± 12.91^a^	5.618	.004
Group B	87.71 ± 14.35			98.48 ± 11.71^b^		
Group C	88.67 ± 13.74			91.8 ± 8.85		
GSES						
Group A	20.63 ± 6.34	0.062	.939	24.79 ± 7.98^a^	3.705	.026
Group B	20.76 ± 6.48			24.33 ± 7.20^b^		
Group C	21.02 ± 5.92			21.13 ± 5.54		

*p* values are based on ANOVA or cross-tabs with Fisher’s exact tests for comparisons across the three resident groups and do not reflect any pairwise comparisons

^a^represents group A vs. group C *p* < .05,

^b^represents group B vs. group C *p* < .05,

^c^represents group A vs. group B *p* < .05

In contrast, when comparing group C with group A (44.49 ± 6.52 vs. 38.18 ± 5.43, *p* < 0.05) and group B (41.75 ± 7.18 vs. 38.18 ± 5.43, *p* < 0.05), significant increases in the post-test scores for perception were found. Moreover, group A’s score was higher than group B’s (44.49 ± 6.52 vs. 0.41.75 ± 7.18, *p* < 0.05). After the intervention, when compared with group C, it was revealed that group A (96.49 ± 12.91 vs. 91.8 ± 8.85, *p* < 0.05) and group B (98.48 ± 11.71 vs. 91.8 ± 8.85, *p* < 0.05) had significantly increased in the post-test scores for attitude. Although the average score of group B was higher than that of group A (98.48 ± 11.71 vs. 96.49 ± 12.91, *p* > 0.05), the difference was not significant. Similar results were found in the post-tests for self-efficacy, with substantial differences in group A (24.79 ± 7.98 vs. 21.13 ± 5.54, *p* < 0.05) and group B (24.33 ± 7.20 vs. 21.13 ± 5.54, *p* < 0.05) group C; however, no significant difference was found between groups A and B (*p* > 0.05).

Univariate and multivariate GEE was used to analyse the variables affecting the cognitive, attitude, and self-efficacy differences of workplace violence among the three groups of subjects before and after they participated in the intervention. Multivariate GEE analyses of factors significant in the univariate analysis were performed, as depicted in Table 4.

**Table 4:** Generalised estimating equations (GEE) analysis

ITEM	POAS			MAVAS			GSES		
	Wald	B	p	Wald	B	p	Wald	B	p
TIME (after vs. before)	46.147	0.433	< .001*	33.039	3.133	< .001*	19.057	0.117	< .001*
Group	17.709	-	< . 001*	6.042	-	.049*	3.19	-	.203
Group A vs. Group C	12.619	3.841	< . 001*	7.661	5.608	.005*	7.124	3.272	.004*
Group B vs. Group C	39.144	6.537	< . 001*	16.523	7.365	< .001*	16.066	3.139	.006*
TIME * group	27.928	-	< . 001*	6.919	-	.031*	11.547	-	.003*
Sex (female vs. male)	-	-	-	8.38	3.769	.004*	3.932	-1.478	.047*
Marital status	9.129	-	.028*	55.955	-	< .001*	13.657	-	.003*
Single vs. widowed	7.208	3.65	.007*	53.584	-12.135	< .001*	13.515	-3.875	< .001*
Unmarried cohabitation vs. widowed	3.047	3.114	.081	12.689	-13.805	< .001*	0.808	-2.433	.369
Married vs. widowed	1.410	2.441	.235	6.472	-11.773	.011*	7.328	-5.384	.007*
Resident	-	-	-	-	-	-	5.187	-	.075
PGY3 vs. PGY1	-	-	-	-	-	-	5.163	2.164	.023*
PGY2 vs. PGY1	-	-	-	-	-	-	0.104	0.262	.746
Work experience	8.914	-	.03*	-	-	-	-	-	-
> 24 months vs. < 6 months	4.059	1.801	.015*	-	-	-	-	-	-
12–24 months vs. < 6 months	1.194	-1.1	.275	-	-	-	-	-	-
6–12 months vs. < 6 months	0.403	0.625	.526	-	-	-	-	-	-
Workplace violence report (yes vs. no)	3.581	1.321	.058	-	-	-	-	-	-
Workplace violence training (yes vs. no)	4.802	1.461	.028*	2.111	1.842	.146	3.383	1.333	.066
Workplace violence exposure (yes vs. no)	5.656	1.717	.017*	0.407	0.829	.524	-	-	-
Physical violence witness (yes vs. no)	-	-	-	1.632	1.694	.201	-	-	-
Workplace violence concern (yes vs. no)	5.677	-	.225	-	-	-	-	-	-

*represents *p*<.05

In the GEE analyses of WPV perceptions, significant differences were found in group, time, group*time interaction, marital status, work experience, WPV training, and WPV exposure (*p* < 0.05). The results showed that the changes in WPV perceptions in the SBME + BE intervention (the average score increased by 6.9, *p* < 0.01) and SBME intervention (the average score increased by 4.67, *p* < 0.01) were better than those in group C.The SBME + BE intervention recorded better results than the SBME intervention only (the average score increased by 2.23, *p* < 0.05). The main differences stem from marital status, work experience, workplace violence training, and WPV exposure. The scores of the participants who were single (B = 3.65, *p* < 0.05) and those who had more than 24 months of work experience had the highest scores (B = 1.801, *p* < 0.05). The scores of the participants who had received WPV training were higher than those without such training (B = 1.461, *p* < 0.05) and those who had experienced WPV were higher than those who had not (B = 1.717, *p* < 0.05).

In the GEE analyses of attitude, significant differences were found in group, time, group*time interaction, sex, and marital status (*p* < 0.05). The results indicate that the changes in attitude in the SBME + BE intervention (the average score increased by 8.02, *p* < 0.01) and SBME intervention (the average score increased by 10.77, *p* < 0.01) were better than those in the control group; however, there were no significant differences between group A and group B. The main differences arose from sex and marital status; the scores of the participants who were widowed had the highest scores (B = -12.135, *p* < 0.01), and females had better scores than males (B = 3.769, *p* < 0.01).

In the GEE analyses of self-efficacy, significant differences were found in time, group*time interaction, sex, marital status, and year of residence. The results indicate that the changes of self-efficacy in group A (the average score increased by 4.16, *p* < 0.01) and group B (the average score increased by 3.57, *p* < 0.01), were better than those in group C. The main differences were related to sex, marital status, and year of residence. The scores of the participants who were widowed were the highest (B = -3.875, *p* < 0.01), males had better scores than females (B = -1.478, *p* < 0.05), and PGY3 residents had better scores than PGY1 residents (B = 2.164, *p* < 0.05).

After the SBME and SBME + BE interventions, the perception, attitude, and self-efficacy pertaining to workplace violence improved significantly. Furthermore, the SBME + BE intervention recorded better scores than the other two groups in the perception of workplace violence.

## Discussion

This study pushed the boundary past previous cross-sectional and quasi-experimental studies without control groups. In addition, we tested a SBME combined with BE model to promote the perception, attitude, and self-efficacy regarding workplace violence among medical residents. The study results of the study reveal statistically significant promotions in perception, attitude, and self-efficacy in coping with WPV after the SBME + BE and SBME-only interventions.Notably,the average score following the SBME + BE interventions was higher than that of the SBME-only intervention in the post-test scores for perception, which can probably be ascribed to the BE effect.

Furthermore, a higher perception of WPV is correlated with single residents, those with more work experience, and those who had prior experiences of WPV training and WPV;a more positive attitude of WPV is correlated with female and widowed residents; and a higher self-efficacy of WPV is correlated with male, widowed, and PGY3 residents.

Several studies in related fields clearly demonstrated that SBME is a realistic but safe and effective method in coping with WPV—it can enhance the medical residents' perception, attitude, and confidence and reduce the incidence of WPV [31–34, 36]. Following two years of education on violence prevention for psychiatrists and residents in outpatient departments, improved effectiveness and confidence in coping with WPV were reported after the training had been completed [75]. A study showed that SBME, combined with role-playing, provides an effective method for violence prevention and effectiveness assessment, and 136 participants reported having acquired a practical skills base and accumulated experiences, which resulted in improved skills for coping with WPV [30]. Some quasi-experimental studies showed that SBME of workplace violence intervention could significantly improve the cognition, attitude, and self-confidence of nurses and student nurses in dealing with WPV [32, 33, 76, 77]. One recent study found that simulation training improved the self-efficacy of aged-care workers and helped to prevent aggressive workplace events [78]. Moreover, a scoping review analysing seven studies on the prevention of WPV through training and simulation education among nurses reported that all those studies confirmed that SBME not only provided skills to prevent injuries from violent incidents, but also improved skills with respect to language degradation and risk assessment. It is suggested that future studies should employ control groups and focus on the long-term effects of interventions [77, 79].

BE is a relatively new field that addresses violence and can assist with the comprehension and control of violence [42]. One recent study indicates that the incidence of intimate violence decreased significantly following the intervention of BE, and the efficacy of the intervention proved to have been sustained at a follow-up assessment four months later [43]. Another study, comprising 623 individuals, found that environmental design plays a significant role in reducing fear of crime and can change people's unacceptable behaviour [45]. In addition, Schulze and Wansink emphasise their viewpoint that BE can provide a better understanding of both stigma and strategies for mitigation [46]. Other studies illustrate the benefits of BE in doctor-patient communication, end-of-life care, and reducing unnecessary conflict [44, 80].

The results of this study of medical residents are similar to those of the aforementioned studies, indicating that both SBME + BE and SBME-only interventions are effective teaching methods to improve the perception, attitude, and self-efficacy of medical residents in coping with WPV. In addition, this study arrived at two novel findings: that SBME + BE results in higher levels of medical residents’ improved perception of WPV, compared with SBME-only interventions. The data support the viewpoint that BE can provide a more effective approach to understanding and managing WPV. Another novel finding is that self-efficacy had improved significantly after the medical residents had been exposed to situational teaching and BE interventions.

This study shows that the majority of medical residents (54.74%) experience WPV in wards frequently. Several previous studies clearly showed that not only are medical residents among the most vulnerable groups to both worker-to-worker and patient-to-worker violence, but WPV against medical residents is also underreported [11, 14, 15]. Our study found that medical residents who had prior experiences of WPV and WPV training scored higher on WPV perception and that prior experiences may have had a positive impact on their ability to cope with WPV. Moreover, those with more work experience had higher scores on WPV perception than those with less than six months’ work experience; it appears that a wealth of clinical experience may be helpful when dealing with WPV. The lower scores of medical residents whose marital status was widowed may have been influenced by mental and physical changes. In other words, the factors of marital status, work experience, WPV training, and WPV exposure can affect the perception of WPV. Acquired knowledge should therefore be integrated with clinical practice, and these influencing factors should be duly considered when designing WPV prevention courses.

In terms of attitudes towards WPV, our research shows that sex and marital status are closely related to attitudes. Compared to male residents, females displayed more positive attitudes in facing workplace aggression. Sex differences in WPV have been reported on in previous studies, with female doctors experiencing less WPV than male doctors [81, 82]. Widowed residents tend to have more positive attitudes than single, married, and unmarried cohabitation residents, possibly due to previous experiences of frustration.

Previous studies indicate that male residents have higher levels of self-efficacy than women [83] and that senior residents’ scores in medical decision-making were higher than those of junior residents [84]. Similar results were found in our study; in terms of self-efficacy to workplace violence, males showed higher self-efficacy in coping with WPV than females did. Moreover, we found that the self-efficacy scores of WPV were higher among PGY3 medical residents than PGY1 residents. Widowed residents displayed higher levels of self-efficacy than the single and married groups, the reason possibly being that the latter groups had stronger willpower. Based on these findings, managers should strengthen residents' self-efficacy in coping with WPV in their first year and provide the necessary assistance to female residents.

This study has several limitations that should be considered. First, the confined geographic area and socio-demographic homogeneity limit the generalisability of the results despite the statistically significant differences between the study groups. Second, the study only examined the source of variation, not the related interactions. However, the focus of this study was to analyse the effect of the intervention and to conduct a preliminary exploration of the variation sources of different intervention effects—the interaction of variation sources should be further explored by future studies. Third, the intervention of ‘behavioural economic education on WPV prevention’ draws more from the psychological analysis of BE and lacks quantitative economic analysis. To the best of our knowledge, this study is the first of its kind to test combined BE and SBME to promote effectiveness among medical residents in coping with WPV. It is therefore suggested that economic analyses should be incorporated in future research. Regardless of its limitations, the study contributes important evidence to the field of WPV prevention approaches and the future exploration of the application of BE in the field of WPV. Further studies should examine the factors affecting the learning effect as well as the long-term influence of BE on intervention outcomes to assess the continued sustainability of these approaches towards WPV.

## Conclusions

This study constructed ‘workplace violence prevention’, ‘simulation education on workplace violence training’, and ‘behavioural economics of workplace violence prevention’, and contributed important evidence regarding the promotion of perception, attitude, and self-efficacy after both SBME + BE and SBME interventions among medical residents in coping with WPV. The highest perception scores were found after the SBME + BE intervention, which could be explained by the inclusion of the BE effect. Hence, we posit that regular simulations and incorporating training on WPV prevention to new medical residents will assist with improving their coping ability, adaptability, and job competence. Further, introducing BE in WPV courses will result in increased knowledge and improved understanding of how to deal with it. Most importantly, these new findings suggest that promoting the application of BE approaches in the field of WPV can reduce and even prevent WPV incidents at work.

## Supplementary Information

**Additional file 1.** Questionnaire Survey onHospital Workplace Violence1.Basicinformation.

## Data Availability

All data generated or analysed during this study are included in this published article.

## Abbreviations

GSES: General self-efficacy scale
MAVAS: Management of aggression and violence attitude scale
POAS: Perception of aggression scale
SBME: Simulation-based medical education
SBME + BE: Simulation-based medical education combined with behavioural economics
PGY: Postgraduate year
WPV: Workplace violence
