# Supplementary material for: A combined behavioural economics- and simulation-based medical education to promote effectiveness among medical residents in coping with workplace violence in Northern China: a quasi-experimental study
Source: BMC Public Health. 2022 Jun 1;22:1090. doi: 10.1186/s12889-022-13497-y (PMC9156828; doi:10.1186/s12889-022-13497-y)
Supplement: Supplementary file 1 — Additional file 1. Questionnaire Survey onHospital Workplace Violence1.Basicinformation. [file 12889_2022_13497_MOESM1_ESM.docx]

Additional file 1:Questionnaire Survey on Hospital Workplace Violence

1.Basic information.

| Medical residents’ sociodemographic characteristics | |
| --- | --- |
| 1 | What is your age? ____________ |
| 2 | What is your gender?  A.Female B.Male |
| 3 | What is your marital status?  A.Single B.Married C.Unmarried cohabitation D.Divorced/widowed |
| 4 | What is your current resident grade?  A.Postgraduate year 1 B. Postgraduate year 2 C.Postgraduate year 3 |
| 5 | Which clinical unit do you currently work in?  A.Pediatrics B. Obstetrics-Gynecology C.Internal medicine D.Neurology E.Surgical F.Radiology |
| 6 | How long is your work experience in hospital?  A.＜6 months B.6-12 months C.12-24 months D.＞24 months E.None |
| 7 | How long do you work in clinical unit every day?  A.＜8 hours B.8-12 hours C.＞12 hours |
| 8 | How worried are you about violence in your current workplace?  A.Absolutely not worried B.A little worried C.Moderately worried D.Worried E.Very worried |
| 9 | Do you know how to report workplace violence?  A.Yes  B.No |
| 10 | Have you received a workplace violence training course?  A.Yes  B.No |
| 11 | Have you ever seen physical violence in workplace?  A.Yes  B.No |
| 12 | Have you ever seen verbal violence in workplace?  A.Yes  B.No |
| 13 | Have you ever experienced from physical violence?  A.Yes  B.No |
| 14 | Have you ever experienced from verbal violence?  A.Yes  B.No |
| 15 | Have you ever experienced from sexual harassment?  A.Yes  B.No |

2.Perception of aggression scale (POAS)

| 12 items | | **Strongly agree** | **Agree** | **Uncertain** | **Disagree** | **Strongly disagree** |
| --- | --- | --- | --- | --- | --- | --- |
| 1 | Aggression is an unpleasant and repulsive behaviour. | **1** | **2** | **3** | **4** | **5** |
| 2 | Aggression is unnecessary and unacceptable. | **1** | **2** | **3** | **4** | **5** |
| 3 | Aggression is hurting others mentally or physically. | **1** | **2** | **3** | **4** | **5** |
| 4 | Aggression is an actual action of physical violence of a patient against a clinicians. | **1** | **2** | **3** | **4** | **5** |
| 5 | Aggression is always negative and unacceptable; feeling should be expressed in another way. | **1** | **2** | **3** | **4** | **5** |
| 6 | Aggression is a disturbing intrusion to dominate others. | **1** | **2** | **3** | **4** | **5** |
| 7 | Aggression is the start of a positive doctor-patient relationship. | **1** | **2** | **3** | **4** | **5** |
| 8 | Aggression is a healthy reaction to feelings of anger. | **1** | **2** | **3** | **4** | **5** |
| 9 | Aggression is an opportunity to get a better understanding of the patient’s situation. | **1** | **2** | **3** | **4** | **5** |
| 10 | Aggression is a form of communication and as such not destructive. | **1** | **2** | **3** | **4** | **5** |
| 11 | Aggression is a way to protect yourself. | **1** | **2** | **3** | **4** | **5** |
| 12 | Aggression is the protection of one’s own territory. | **1** | **2** | **3** | **4** | **5** |

3.Management of aggression and violence attitude scale (MAVAS)

| 27 items | | **Strongly agree** | **Agree** | **Uncertain** | **Disagree** | **Strongly disagree** |
| --- | --- | --- | --- | --- | --- | --- |
| 1 | Improved one to one relationships between staff and patients can reduce the incidence of patient aggression and violence. | **1** | **2** | **3** | **4** | **5** |
| 2 | The use of deescalation is successful in preventing violence. | **1** | **2** | **3** | **4** | **5** |
| 3 | The use of negotiation could be used more effectively when managing aggression and violence. Aggressive patients will calm down automatically if left alone. | **1** | **2** | **3** | **4** | **5** |
| 4 | Patient aggression could be handled more effectively on this ward. | **1** | **2** | **3** | **4** | **5** |
| 5 | The use of negotiation could be used more effectively when managing aggression and violence. Aggressive patients will calm down automatically if left alone. | **1** | **2** | **3** | **4** | **5** |
| 6 | If the physical environment were different, patients would be less aggressive. | **1** | **2** | **3** | **4** | **5** |
| 7 | It is largely situations that contribute towards the expression of aggression by patients. | **1** | **2** | **3** | **4** | **5** |
| 8 | Physical restraint is sometimes used more than necessary. | **1** | **2** | **3** | **4** | **5** |
| 9 | Alternatives to the use of containment and sedation to manage patient violence could be used more frequently. | **1** | **2** | **3** | **4** | **5** |
| 10 | Seclusion is sometimes used more than necessary. | **1** | **2** | **3** | **4** | **5** |
| 11 | The practice of secluding violent patients should be discontinued. | **1** | **2** | **3** | **4** | **5** |
| 12 | Expressions of aggression do not always require staff intervention. | **1** | **2** | **3** | **4** | **5** |
| 13 | Restrictive care environments can contribute towards patient aggression and violence. | **1** | **2** | **3** | **4** | **5** |
| 14 | Prescribed medication can in some instances lead to patient aggression and violence. | **1** | **2** | **3** | **4** | **5** |
| 15 | Poor communication between staff and patients leads to patient aggression. | **1** | **2** | **3** | **4** | **5** |
| 16 | Patients commonly become aggressive because staff do not listen to them. | **1** | **2** | **3** | **4** | **5** |
| 17 | Other people make patients aggressive or violent. | **1** | **2** | **3** | **4** | **5** |
| 18 | Patients are aggressive because of the environment they are in. | **1** | **2** | **3** | **4** | **5** |
| 19 | Patients are aggressive because they are ill. | **1** | **2** | **3** | **4** | **5** |
| 20 | All patients are verbally abusive. | **1** | **2** | **3** | **4** | **5** |
| 21 | Patients who are violent are often restrained for their own safety. | **1** | **2** | **3** | **4** | **5** |
| 22 | It is difficult to prevent patients from becoming violent or aggressive. | **1** | **2** | **3** | **4** | **5** |
| 23 | There appear to be types of patients who frequently become aggressive towards staff. | **1** | **2** | **3** | **4** | **5** |
| 24 | Different approaches are used on this ward to manage patient aggression and violence. | **1** | **2** | **3** | **4** | **5** |
| 25 | Medication is a valuable approach for treating aggressive and violent behaviour. | **1** | **2** | **3** | **4** | **5** |
| 26 | When a patient is violent, seclusion is one of the most effective approaches to use. | **1** | **2** | **3** | **4** | **5** |
| 27 | Patients who are aggressive towards staff should try to control their feelings. | **1** | **2** | **3** | **4** | **5** |

4.The General Self-Efficacy Scale (GSE)

| 10 items | | **Not at all true** | **Hardly true** | **Moderately true** | **Exactly true** |
| --- | --- | --- | --- | --- | --- |
| 1 | I can always manage to solve difficult problems if I try hard enough. | **1** | **2** | **3** | **4** |
| 2 | If someone opposes me, I can find the means and ways to get what I want. | **1** | **2** | **3** | **4** |
| 3 | It is easy for me to stick to my aims and accomplish my goals. | **1** | **2** | **3** | **4** |
| 4 | I am confident that I could deal efficiently with unexpected events. situations. | **1** | **2** | **3** | **4** |
| 5 | Thanks to my resourcefulness, I know how to handle unforeseen. | **1** | **2** | **3** | **4** |
| 6 | I can solve most problems if I invest the necessary effort. | **1** | **2** | **3** | **4** |
| 7 | I can remain calm when facing difficulties because I can rely on my coping abilities. | **1** | **2** | **3** | **4** |
| 8 | When I am confronted with a problem, I can usually find several solutions. | **1** | **2** | **3** | **4** |
| 9 | If I am in trouble, I can usually think of a solution. | **1** | **2** | **3** | **4** |
| 10 | I can usually handle whatever comes my way. | **1** | **2** | **3** | **4** |
